# Ambulatory Surgery for Perianal Crohn's Disease: Study of Feasibility

**DOI:** 10.1155/2018/5249087

**Published:** 2018-12-23

**Authors:** S. Sibio, A. Di Giorgio, M. Campanelli, S. Di Carlo, A. Divizia, C. Fiorani, R. Scaramuzzo, C. Arcudi, G. Del Vecchio Blanco, L. Biancone, G. Sica

**Affiliations:** ^1^Department of Surgery “Pietro Valdoni”, Sapienza University of Rome, Via Lancisi 2, 00155 Rome, Italy; ^2^Policlinico Tor Vergata, Department of Surgery, University Tor Vergata, Viale Oxford 81, 00133 Rome, Italy; ^3^Policlinico Tor Vergata, Department of Medicine, University Tor Vergata, Viale Oxford 81, 00133 Rome, Italy

## Abstract

**Background:**

One-third of Crohn's disease (CD) patients present perianal fistula. The gold standard in the diagnosis and treatment of symptomatic perianal disease (PAD) in CD is the exploration of the anal canal and distal rectum under anesthesia (EUA). This procedure is mainly conducted as a day case surgery. Unfortunately, it is not always possible to proceed within the ideal timing and any delay may well represent a relevant clinical issue. The aim of this study was to evaluate the feasibility of outpatient treatment of symptomatic perianal fistulas in CD patients.

**Methods:**

All CD patients under regular follow-up at our inflammatory bowel disease referral center, presenting with symptomatic perianal fistulas, were offered surgical consultation. The data of patients were prospectively collected for three years (February 2014 to February 2017) for the purpose of the study. All clinical information, including previous EUA and/or records from MRI and endoscopic ultrasound, was included. Outpatient anal canal and distal rectum exploration and treatment (OE) were undertaken during the specialist surgical consultation. Fistulas were classified according to Parks's classification; the type of outpatient treatment and compliance of patients were recorded. Pain was assessed by VAS at the time of the procedure and during the first control. Patients were followed up in the surgical clinic in relation to the study.

**Results:**

Ninety-two CD patients with symptomatic perianal fistulas had surgical consultation during the study period. OE was offered to all but 18 patients who fulfilled the exclusion criteria or had an extremely severe disease; six patients refused the OE (8.11%). Of the 68 patients undergoing OE, eleven (16.18%) had previous surgery for perianal disease. The OE was accomplished in sixty-one patients (89.71%), while in 7 patients, it was abandoned for scarce compliance. Nine patients (14.75%) underwent drainage of perianal abscess; in 3 of them, it was possible to probe the fistula tract, find the internal orifice, and pass a loose seton. Overall, setonage was performed in 50 patients (81.97%). Rectovaginal setons were placed in 3 patients and more than one seton (up to 3) in 6 cases. Fistulotomy was performed in 4 simple subcutaneous fistulous tracts. Concordance with the preoperative findings was found in 54 out of 61 patients. EUA was scheduled at the time of OE for the 7 patients who did not complete the procedure. All sixty-one patients who had the OE were followed up for a minimum of 12 months.

**Conclusions:**

This preliminary study indicates that OE in CD patients with symptomatic perianal fistulas is safe and feasible in a high-volume referral center. It might provide several benefits, including patients' logistics, reduce or remove patients' symptoms and discomfort, allow for a timely start of medical therapy, and avoid further complications.

## 1. Introduction

One-third of Crohn's disease (CD) patients have perianal disease [1–3]. The gold standard to assess symptomatic perianal disease (PAD) in CD patients is the exploration of the anal canal and distal rectum under anesthesia (EUA) [4–7]. EUA usually allows a correct diagnosis of fistulous tracts, a classification of the fistula, and an appropriate treatment of the PAD at the same time. The aim of the surgical therapy is to control sepsis, preserving anorectal functions and avoiding further complications.

The aim of this preliminary study was to assess the feasibility of an outpatient examination (OE) and treatment in a nonselected population of CD patients with symptomatic perianal fistulas.

## 2. Methods

During the study period, all the patients on regular follow-up at our referral center for inflammatory bowel disease (IBD), presenting with symptomatic perianal fistulas, were enrolled in this study of feasibility. There were no general exclusion criteria, but minor legal age. Patients with a known horse-shoe fistula, recorded incontinence, multiple external orifices, substantial discharge, marked discomfort or limitation, and substantial induration or large abscesses were excluded. To assess the severity of the condition, the Perianal Disease Activity Index (PDAI) was used [8]. Furthermore, noncompliant patients or patients with major comorbidities were also excluded. Diagnosis of CD was made according to the conventional clinical, endoscopic, histological, and radiological criteria. A dedicated gastroenterologist and surgeon evaluated all patients. Magnetic resonance imaging (MRI) and/or endoscopic ultrasound examination were performed whenever possible prior to the multidisciplinary consultation. Data regarding demographics and preoperative clinics (including duration of the disease, localization and behavior of CD, previous surgery, comorbidities, current therapy, and radiological or endoscopic assessment of perianal fistulas) were reviewed focusing on the results of previous imaging and surgical reports.

The need for the EUA was explained, and the OE was offered alternatively and, upon a patient's acceptance, done within 48-60 hrs. Informed consent was obtained from all patients. No ethics committee agreement was requested. The OE was conducted with the patient on a left lateral position (same as for any anorectal inspection) always by the same surgeon with the aid of a dedicated specialist nurse. A careful assessment of the perianal region was undertaken in search for lumps, discharge, or orifices. A 9 mm-in-length lighted surgical proctoscope was used to evaluate the anal canal and distal rectum. Small-to-medium size abscesses were drained after local injection of lidocaine 2%+naropine 7.5%. Electrocautery was rarely used to obtain hemostasis, but gentle package with soaked sponges. The external orifice or the abscess cavity was injected with hydrogen peroxide (H_2_O_2_) or a saline solution, to find the internal orifice; an olive-tip malleable metal wire was used to probe the sinus tract. The classification proposed by Parks in 1976 [9] was used for a fistula-type recording. Usually, whenever the internal orifice was found, a loose silicon seton (vessel loop) was placed and secured with a double-silk tie. Occasionally, simple posterior fistulas were treated with open fistulotomy or by means of cutting setons.

In order to assess the feasibility of the outpatient procedure, the following parameters were taken into account. Discomfort or pain was evaluated at the end of the procedure (perioperative pain/discomfort) and after one week, using the Visual Analogical Score (VAS) [10]. The time to discharge (ranging from minutes to hours after the procedure), the need for pain killer therapy, recurrence at one year, and the incidence of anal incontinence were recorded data. In patients who had a cutting seton placed, a weekly outpatient control was scheduled until the fistulotomy was completed. In case of treatment not performed or considered incomplete (i.e., abscess drainage but fistulous tract not found), a day surgery EUA was scheduled and the case was recorded as treatment failure. The data were collected on a Microsoft® Excel spreadsheet, and the results were compared with those available in literature in order to establish if OE may be considered a safe and effective procedure. The evaluation of the late postoperative discomfort and patients' satisfaction was performed by means of a telephone interview.

## 3. Results

Ninety-two consecutive patients with evidence of symptomatic perianal fistulas were seen by our IBD referral center from February 2014 to February 2017. Eighteen patients did not fulfill the inclusion criteria and were excluded from the study: two had horse-shoe fistulas, nine had a PDAI > 3 concerning the number of fistulas, discomfort or pain, and substantial induration or large abscesses, one patient was excluded because of minor legal age, four were excluded because of known incontinence, and two were excluded because they had taken double antiaggregant therapy for cardiac stenting. Compliance was good, as only six patients refused the proposed procedure (8.11%). Of the 68 patients undergoing OE, eleven (16.18%) had already undergone surgery for perianal disease in the past. OE was not completed in seven patients mostly due to discomfort or anxiety during the procedure. In 61 patients (89.71%), it was possible to complete the outpatient exploration of the anal canal and distal rectum to assess the PAD. A surgical treatment was done in 60 of them. No painkillers were given before or just after the procedure.

The patients' characteristics are summarized in Table 1.

The findings in accordance with Parks's classification and surgical treatment are shown in Table 2. Hughes classification is also reported for the purpose of the study [11].

Nine patients (14.75%) underwent drainage of perianal abscess; in 3 of them, it was possible to probe the fistula tract, find the internal orifice, and pass a loose seton. Overall, setonage was performed in 50 patients (81.97%). More than one seton (up to 3) was passed in 6 cases. Fistulotomy was performed in four simple low posterior anorectal sinuses with no involvement or a very subtle involvement of the internal sphincter. In the other ten extrasphincteric fistulas, it was preferred to pass a loose seton, giving the presence of a degree 2-3 discharge (PDAI) or in the presence of a localization in the upper quadrants of the anus. Seven patients subsequently had a cutting seton fistulotomy. Three patients with rectovaginal fistulas had a loose seton through the vaginal orifice. In four cases of very low posterior transsphincteric fistula, fistulotomy was achieved by means of a cutting seton. Concordance with the preoperative findings was found in 54 out of 61 patients (88.52%).

The pain evaluation score showed a median VAS of 2.0 (range 0-4) immediately after the procedure (perioperative) and a median score of 1.0 (range 0-2) a week later.

No complications were recorded during or after the procedures.

Patients left the outpatient department from few minutes to two hours after the procedure with paracetamol (up to 3 grams per day) prescribed for the first 3 days in case of pain.

The first control was scheduled in one week or after 3 days for those patients who had abscess drainage.

All the patients were followed up for a minimum of 12 months. In 39 patients, data from a 24-month follow-up were recorded.

During the FU period, 7 patients (12%) had PAD relapse (requiring intervention):

- Two patients with extrasphincteric fistulas: the first, who had a simple setonage because of severe proctitis, showed a rectovaginal orifice at 8 months (it was in fact a relapse of a previously treated rectovaginal fistula) and underwent EUA and setonage. In the other patient, who had a fistulotomy during the OE, a redo fistulotomy with conization under narcosis was performed to avoid sepsis
- Two cases of suprasphincteric fistula: one patient develops an abscess 5 months from seton placement, came to the emergency department, and had surgical drainage and further setonage under narcosis. The second patient that also had a simple setonage during the OE was hospitalized after 11 months because of severe proctitis, cellulitis, and perineal pain; he underwent EUA, partial fistulectomy, and setonage of a horse-shoe fistula
- In three cases (2 intersphincteric and 1 transsphincteric), it was possible to repeat the outpatient exploration to treat the recurrence and a setonage of a different sinus tract was performed

Furthermore, both the patients with rectovaginal fistulas eventually had in-hospital surgery: in 1 case, a rectal advancement flap was performed, while fistulectomy, repair of the vagina, and a setonage of an adjacent perianal fistula were carried out in the second patient.

At the telephone interview, 57 (95%) patients were highly satisfied of the received treatment, scoring 9 of 10 in a scale 0-10.

## 4. Discussion

Almost one-third of CD patients show symptoms of PAD [10, 12]. The risk of developing PAD is consistent with the time from the diagnosis of CD, 20% after ten years and up to 30% after twenty years. However, PAD is far more common in patients with colon (41%) and rectum (90%) localization and less in patients with ileal disease (12%). [13, 14]. Early diagnosis and correct treatment are crucial to allow patients to promptly start medical treatments with antitumor necrosis factor (tnf) which is considered the cornerstone of treatment, offering the best long-term control of PAD [15, 16]. The diagnosis and treatment may be delayed since the clinical pathway for this subset of patients is still not fully standardized, even in specialized centers. Recently, a delay in many elements of the clinical pathway in patients with Crohn's anal fistula has been reported [15], and it was seen that the commencement of the anti-tnf therapy could be as long as one year. Resolving the delay is also important to reduce the debility associated with PAD.

Perianal fistulas in CD may be simple or complex according to the American Gastroenterological Association (AGA) [17–20]. Simple fistulas have a high healing rate, while complex fistulas are difficult to treat and show a poor healing rate and increased rate of relapse. The proper surgical choice depends on the anatomy, the type of fistula, and finally, the surgeon's expertise [21–25]. Active proctitis control must be achieved whenever possible prior to any surgical treatment. Treatment strategy and procedures are different in an acute or in an elective setting; in acute management, the main aim is sepsis control: incision and drainage of every abscess are strongly advised, while placement of a loose seton should be considered only if the fistulous tract can be promptly and easily identified [7]. In an elective setting, an exploration of the anal canal and distal rectum under anesthesia is recommended and, in case of complex fistula, even in the presence of proctitis, a loose, draining seton could be passed if the internal and external orifices of the fistulous tract are found. A fistulotomy or fistulectomy can be safely considered for simple posterior fistulas [26].

Several operations have been deployed to treat complex perianal fistulas in Crohn's disease. The fistulous tract can be excised, injected with glue, tied and divided, covered with a mucosal flap, or injected with autologous stem cells. Unfortunately, none of these procedures were capable to show a clear advantage over the others, especially in Crohn's disease. The aim of the surgical treatment of PAD in CD patients should be symptoms or complication control, allowing patients to pursue a timely medical therapy, in a multidisciplinary management.

In the presence of a symptomatic perianal fistula, an optimal result can be considered to avoid sepsis, allowing for a good drainage before thinking to the complete healing of the fistula and finally preventing the recurrence and preserving the continence of the anal sphincter. It is essential to ensure timely treatment, because perianal fistulas significantly impair the quality of life of the patients, to avoid the potentially disastrous consequences such as those of an undrained sepsis or ramification of the fistulous tracts.

Only the patients with symptomatic Crohn's anal fistula should undergo a surgical treatment.

The gold standard treatment for symptomatic perianal disease in CD patients is conducted during the EUA. Most of the series available in literature refer to day surgery or overnight admission. Unfortunately, a timely treatment is not always possible and this, as said, may well represent a relevant clinical issue. Our study was designed to evaluate the feasibility of outpatient treatment of CD patients with perianal fistula in terms of safety and feasibility. Patient compliance, clinical results, and costs were also evaluated.

The OE was feasible in the vast majority of patients (74 out of 92, 80.4%), and we had no immediate or delayed complications due to the procedure. Patients' compliance for both procedure acceptance and procedure execution was very high (68 out of 74, 91.9%, and 61 out of 68, 89.7%, respectively).

Twenty-four percent of our patients had colonic or rectal localization of Crohn's disease. These data seem to differ from those of literature (up to 41% in colonic localization and up to 92% in rectal localization) [22], but these data referred to active disease, while in our series, only two patients showed severe active proctitis at OE. It is possible that there was a different method or judgement and that this may introduce a bias in the results. Thirty-seven (59.7%) patients were off therapy at the time of the first perianal symptoms, while 24 (39.3%) were under treatment with immunosuppressive or biological therapy.

In this preliminary series, we recorded the surgical treatment associated with the OE: a total of 50 loose seton placements, 9 abscess drainages, and 4 fistulotomies were performed in 68 patients.

We reported a relatively high rate of seton placement, even in the more difficult fistula types, up to 67% in suprasphincteric fistulas and up to 71% in extrasphincteric ones. According to the Association of Coloproctology of Great Britain and Ireland consensus conference on surgical management of fistulating perianal Crohn's disease, experienced surgeons should always try to place a seton when the fistulous tract is readily identifiable (evidence level 1A) and this should be possible most of the times in “skilled hands” [7]. Moreover, in this series, more than 50% of patients with extrasphincteric or suprasphincteric fistulas had been studied by means of MRI before the OE.

Seven patients relapse one year after the OE (10.9%). Nonetheless, those are not real relapses, considering that the aim of the treatment was not the healing of the fistulous tract. However, it was necessary to take these patients back to undergo a subsequent operation. No alternative treatment to obturate the fistulous tract was proposed in the two-year study period. We believe that stem cell therapy according to the ADMIRE-CD trial [27] can be proposed in the outpatient setting.

In this series, OE seems feasible with good results at one year. Compliance of patients to the procedure was high and, from a surgical point of view, the OE was nice to perform without difficulties or trouble in all cases. In this series, there was never a case in which the surgeon felt to be in a difficult situation. The OE was abandoned only in few cases, mostly of high transsphincteric or suprasphincteric fistulas, due to the patients' discomfort. Horse-shoe fistulas do represent a limit to OE.

The aim of the OE was anal canal exploration and surgical control of patients' symptoms and prevention of further complications related to Crohn's perianal fistulas.

We believe that OE could be an efficacious answer to the issues recently underlined by Lee et al. [15] and those reported above. OE can be a part of a clinical pathway for patients with perianal Crohn's fistulas. It is perceived by patients as less invasive and allows for timely commencement of anti-tnf therapy. This is, in the authors' opinion, [3] the best clinical scenario, since Crohn's disease is a systemic degenerative chronic condition in which the perianal disease represents only a local, although troublesome, occurrence for some patients. A less invasive procedure, capable of minimizing distress and discomfort for patients often deemed to several major and minor surgical procedures during their life and in whom a timely medical treatment, is crucial.

This procedure should be offered in a high-volume center in which a multidisciplinary dedicated team is available. In selected cases, OE may be offered as a “bridge to surgery,” able to faster solve critical clinical issues or palliate disabling symptoms with low morbidity and discomfort, also allowing patients to continue medical therapy. OE can be repeated, if necessary, in different occasion. From an economical point of view, the OE can definitively save logistics and money. This can also represent a relevant point in favour of the OE, considering that, as reported by a Spanish survey, the yearly costs of CD patients for the national health system account for 8.289 euros per patient and that 12.4% of this goes for surgical treatment [28].

The OE surely is a minimally invasive approach, with low morbidity and very low patient stress. It is the authors' opinion that the key success of the OE is the relationship of the patients with the referral IBD center. In this series, all the patients were known to the center and the multidisciplinary consultation plays a great role for increasing patients' compliance. Our study suggests that OE could be a safe and effective procedure that can be proposed to the vast majority of patients with Crohn's fistulas. It is not recommended in nonexperienced hands and in high complex or rectovaginal fistulas (Hughes classification 1b, 1d or 2d, and 2e). OE could be compared to EUA to assess the perceived advantages of this preliminary case series.

## Figures and Tables

**Table 1:** Patients' characteristics.

Characteristics	
Sex ratio (M : F)	35 : 26
Age (median, years)	32.8 (18-56)
Duration of perianal disease: median (months)	17 (0-68)

Localization of CD (%)	Distal ileum and ascending colon (65%) Left colon/rectum (24%) Other localizations (11%)

Current therapy	Antibiotics +/- other medications: 30 (49.18%)
	Steroids only: 16 (26.23%)
	5-ASA: 2 (3.28%)
	Thiopurine: 4 (6.56%)
	Infliximab or similar: 4 (6.56%)

Previous perianal surgery	11 (16.18%)

Imaging	Yes: 20 MRI: 20 (32.79%) MRI + US: 5 (8.19%) No: 41

**Table 2:** Results of our study according to Parks' (Hughes) classification.

Parks' type of fistula (Hughes cl.).	No. (%)	Type of treatment no. (%)				VAS (median 0-10)	Relapse after procedure at 1 year (%)	Imaging (no. of exams)		Concordance with imaging	
		Setonage	Fistulotomy	Drainage	No treatment			MRI	US	MRI	US
Suprasphincteric (2a)	9 (14.75%)	6	0	3	0	1.57 (0-3)	2 (22.22%)	3	1	1/3	1/1
Intersphincteric (1c)	25 (40.98%)	23	0	3	0	2.25 (0-4)	2 (8%)	2	0	2/2	
Extrasphincteric (2b)	14 (22.95%)	10	4	2	0	1.7 (1-3)	2 (14.29%)	8	0	6/8	
Transsphincteric	8 (13.11%)	8	0	0	0	1.7 (0-4)	1 (12.5%)	3	2	2/3	2/2
Rectovaginal (2d)	3 (4.92%)	3	0	0	0	2.5 (0-3)	0	2	2	2/2	2/2
Unclassified	2 (3.28%)	0	0	1	1	4 (1-4)	0	2	0	0/2	
Tot	61	50	4	9	1		6	20	5		

## Data Availability

The data used to support the findings of this study are included within the article.

## References

[1] Chung W., Ko D., Sun C., Raval M. J., Brown C. J., Phang P. T. (2010). Outcomes of anal fistula surgery in patients with inflammatory bowel disease. *The American Journal of Surgery*.

[2] Schwartz D. A., Loftus E. V., Tremaine W. J. (2002). The natural history of fistulizing Crohn’s disease in Olmsted County, Minnesota. *Gastroenterology*.

[3] Sica G. S., di Carlo S., Tema G. (2014). Treatment of peri-anal fistula in Crohn’s disease. *World Journal of Gastroenterology*.

[4] Haggett P. J., Moore N. R., Shearman J. D., Travis S. P., Jewell D. P., Mortensen N. J. (1995). Pelvic and perineal complications of Crohn’s disease: assessment using magnetic resonance imaging. *Gut*.

[5] Buchanan G. N., Halligan S., Bartram C. I., Williams A. B., Tarroni D., Cohen C. R. G. (2004). Clinical examination, endosonography, and MR imaging in preoperative assessment of fistula in ano: comparison with outcome-based reference standard. *Radiology*.

[6] Sloots C. E., Felt-Bersma R. J., Poen A. C., Cuesta M. A., Meuwissen S. G. (2001). Assessment and classification of fistula-in-ano in patients with Crohn’s disease by hydrogen peroxide enhanced transanal ultrasound. *International Journal of Colorectal Disease*.

[7] Lee M. J., Heywood N., Sagar P. M., Brown S. R., Fearnhead N. S., the ACPGBI Perianal Crohn's Disease Group (2017). Association of Coloproctology of Great Britain and Ireland consensus exercise on surgical management of fistulating perianal Crohn’s disease. *Colorectal Disease*.

[8] Irvine E. J. (1995). Usual therapy improves perianal Crohn’s disease as measured by a new disease activity index. McMaster IBD Study Group. *Journal of Clinical Gastroenterology*.

[9] Parks A. G., Gordon P. H., Hardcastle J. D. (1976). A classification of fistula-in-ano. *British Journal of Surgery*.

[10] Huskisson E. C. (1974). Measurement of pain. *The Lancet*.

[11] Hughes L. E. (1992). Clinical classification of perianal Crohn’s disease. *Diseases of the Colon & Rectum*.

[12] Singh B., McC. Mortensen N. J., Jewell D. P., George B. (2004). Perianal Crohn’s disease. *British Journal of Surgery*.

[13] van der Hagen S. J., Baeten C. G., Soeters P. B., van Gemert W. G. (2006). Long-term outcome following mucosal advancement flap for high perianal fistulas and fistulotomy for low perianal fistulas: recurrent perianal fistulas: failure of treatment or recurrent patient disease?. *International Journal of Colorectal Disease*.

[14] Scanu A. M. (2008). *Fistole perianali nella malattia di crohn iter formativo in coloproctologia corso avanzato e update in coloproctologia Vercelli*.

[15] Lee M. J., Freer C., Adegbola S. (2018). Patients with perianal Crohn’s fistulas experience delays in accessing anti-TNF therapy due to slow recognition, diagnosis and integration of specialist services: lessons learned from three referral centres. *Colorectal Disease*.

[16] Gold S. L., Cohen-Mekelburg S., Schneider Y., Steinlauf A. (2018). Perianal fistulas in patients with Crohn’s disease, part 1: current medical management. *Gastroenterology & Hepatology*.

[17] Ommer A., Herold A., Berg E. (2017). German S3 guidelines: anal abscess and fistula. *Langenbeck's Archives of Surgery*.

[18] Koelbel G., Schmiedl U., Majer M. C. (1989). Diagnosis of fistulae and sinus tracts in patients with Crohn disease: value of MR imaging. *AJR American Journal of Roentgenology*.

[19] Bouchard D., Abramowitz L., Bouguen G. (2017). Anoperineal lesions in Crohn’s disease: French recommendations for clinical practice. *Techniques in Coloproctology*.

[20] Orsoni P., Barthet M., Portier F., Panuel M., Desjeux A., Grimaud J. C. (1999). Prospective comparison of endosonography, magnetic resonance imaging and surgical findings in anorectal fistula and abscess complicating Crohn’s disease. *British Journal of Surgery*.

[21] Solomon M. J., McLeod R., O’Connor B. I., Steinhart A. H., Greenberg G. R., Cohen Z. (1993). Combination of ciprofloxacin and metronidazole in severe perianal Crohn’s disease. *Canadian Journal of Gastroenterology*.

[22] Al-Khawari H. A., Gupta R., Sinan T. S., Prakash B., Al-Amer A., Al-Bolushi S. (2005). Role of magnetic resonance imaging in the assessment of perianal fistulas. *Medical Principles and Practice*.

[23] Sandborn W. J., Fazio V. W., Feagan B. G., Hanauer S. B., American Gastroenterological Association Clinical Practice Committee (2003). AGA technical review on perianal Crohn’s disease. *Gastroenterology*.

[24] Gecse K. B., Bemelman W., Kamm M. A. (2014). A global consensus on the classification, diagnosis and multidisciplinary treatment of perianal fistulising Crohn’s disease. *Gut*.

[25] Kotze P. G., Shen B., Lightner A. (2018). Modern management of perianal fistulas in Crohn’s disease: future directions. *Gut*.

[26] Bemelman W. A., Warusavitarne J., Sampietro G. M. (2018). ECCO-ESCP consensus on surgery for Crohn’s disease. *Journal of Crohn's and Colitis*.

[27] Panés J., García-Olmo D., van Assche G. (2018). Long-term efficacy and safety of stem cell therapy (Cx601) for complex perianal fistulas in patients with Crohn’s disease. *Gastroenterology*.

[28] Chaparro M., Zanotti C., Burgueño P. (2013). Health care costs of complex perianal fistula in Crohn’s disease. *Digestive Diseases and Sciences*.

